# Prevalence and Characteristics of Arthritis Among Caregivers — 17 States, 2017 and 2019

**DOI:** 10.15585/mmwr.mm7144a1

**Published:** 2022-11-04

**Authors:** Eva M.J. Jackson, John D. Omura, Michael A. Boring, Erica L. Odom, Anika L. Foster, Benjamin S. Olivari, Lisa C. McGuire, Janet B. Croft

**Affiliations:** ^1^Alzheimer’s Association, Chicago, Illinois; ^2^Oak Ridge Institute for Science and Education, Oak Ridge, Tennessee; ^3^Division of Population Health, National Center for Chronic Disease Prevention and Health Promotion, CDC; ^4^ASRT, Inc., Smyrna, Georgia.

Caregiving provides numerous benefits to both caregivers and care recipients; however, it can also negatively affect caregivers’ mental and physical health (*1*–*4*), and caregiving tasks often require physical exertion (*1*). Approximately 44% of adults with arthritis report limitations attributable to arthritis, including trouble doing daily activities (*5*). These limitations might affect caregivers’ ability to provide care, but little is known about arthritis among caregivers. To assess arthritis among caregivers of a family member or friend, CDC examined data from 17 states that administered both the arthritis and caregiving modules as part of the Behavioral Risk Factor Surveillance System (BRFSS) in either 2017 or 2019. Approximately one in five adults (20.6%) was a caregiver. Prevalence of arthritis was higher among caregivers (35.1%) than noncaregivers (24.5%). Compared with caregivers without arthritis, those with arthritis provided similar types of care and were more likely to have provided care for ≥5 years and for ≥40 hours per week. In addition, higher proportions of caregivers with arthritis reported disabilities compared with those without arthritis, including mobility issues (38.0% versus 7.3%). Arthritis among caregivers might affect their own health as well as the care they can provide. Caregivers can discuss their arthritis and related limitations with a health care professional to identify ways to increase their physical activity and participation in lifestyle management programs.* Such interventions might ease arthritis pain and related limitations and might support them in their ongoing caregiving role. Public health professionals can implement strategies to support caregivers throughout the caregiving process.^†^

BRFSS is a cross-sectional, random-digit–dialed, annual telephone survey of noninstitutionalized U.S. adults aged ≥18 years. BRFSS is conducted by state and territorial health departments, and data are weighted to make estimates representative of each state. BRFSS data were analyzed among 17 states^§^ using the most recent year (2017 or 2019) in which respondents were asked both the caregiving and arthritis module questions (including arthritis-related limitation questions) in the same year. Combined (landline and mobile) median response rates for states used in the analysis were 47.3% (2017) and 45.7% (2019).^¶^

Respondents were classified as caregivers of a family member or friend if they responded “yes” when asked whether they provided care to a family member or friend with a health condition or disability during the past 30 days. Respondents were classified as having arthritis if they responded “yes” when asked if they had ever been told by a doctor or other health professional that they have some form of arthritis, rheumatoid arthritis, gout, lupus, or fibromyalgia. Data were collected from 106,316 respondents; 15,195 (14.3%) respondents who refused to respond, who responded “don’t know/not sure,” or were missing responses on either the caregiving or arthritis question were excluded from the analysis. The final sample size included 91,121 respondents.

Prevalence of arthritis was compared between caregivers and noncaregivers overall and by selected demographic subgroups and individual states. Bivariate analyses were conducted among caregivers with and without arthritis to assess distributions of characteristics related to caregiving (length of care,** weekly hours of care,^††^ and type of care provided^§§^), having a primary care provider, and status of disability types that might be related to arthritis (mobility, self-care, and independent living disabilities).^¶¶^ Among caregivers with arthritis, prevalence of arthritis-attributable activity and work limitations*** was determined. Distribution of employment status^†††^ was determined among caregivers providing ≥40 hours of caregiving per week by arthritis status to examine employment status among those who provide care full-time. Analyses were conducted using SUDAAN (version 11.0; RTI International) to account for the complex survey design and weighting. Statistical significance was determined at α = 0.05. This activity was reviewed by CDC and was conducted consistent with applicable federal law and CDC policy.^§§§^

In 17 states during 2017 and 2019, one in five adults (20.6%; 95% CI = 20.1%–21.2%) was a caregiver, and more than one in three (35.1%) caregivers had arthritis (Table 1). Prevalence of arthritis was greater among caregivers than among noncaregivers overall (35.1% versus 24.5%), in each state, and across all demographic subgroups by age group, sex, education status, body mass index category, and inactivity status. Prevalence of arthritis was higher among caregivers than among noncaregivers for most employment statuses, races, and ethnicities.

**TABLE 1:** Prevalence of arthritis* among caregivers^†^ and noncaregivers aged ≥18 years, by selected characteristics and state — Behavioral Risk Factor Surveillance System, 17 states,^§^ 2017 and 2019

Characteristic	Caregivers		Noncaregivers		p-value^††^
	Unweighted no.^¶^	Prevalence of arthritis,** % (95% CI)	Unweighted no.^¶^	Prevalence of arthritis,** % (95% CI)	
**Overall**	**19,910**	**35.1 (33.8–36.5)**	**71,211**	**24.5 (23.9–29.1)**	**<0.001**
**Age group, yrs**					
18–44	4,207	17.3 (15.2–19.6)	19,453	7.8 (7.2–8.5)	<0.001
45–64	8,215	39.1 (37.1–41.2)	24,202	31.5 (30.3–32.7)	<0.001
≥65	7,240	55.4 (53.2–57.6)	26,685	49.5 (48.2–50.7)	<0.001
**Sex**					
Men	7,419	30.6 (28.6–32.7)	32,967	20.9 (20.0–21.7)	<0.001
Women	12,488	38.4 (36.7–40.1)	38,230	28.1 (27.3–29.1)	<0.001
**Race and ethnicity**					
American Indian or Alaska Native, non-Hispanic	462	34.8 (23.7–47.8)	1,363	26.2 (21.4–31.5)	0.20
Asian, non-Hispanic	495	24.1 (15.5–35.5)	2,692	10.0 (7.5–13.1)	<0.001
Black or African American, non-Hispanic	1,315	29.5 (25.3–34.2)	4,460	26.2 (24.1–28.4)	0.19
White, non-Hispanic	14,802	38.7 (37.2–40.2)	51,328	27.9 (27.2–28.6)	<0.001
Hispanic	1,463	22.1 (18.5–26.2)	6,851	14.8 (13.3–16.4)	<0.001
Other, non-Hispanic^§§^	789	35.2 (28.7–42.4)	2,450	26.2 (22.7–30.2	0.02
**Education level**					
High school graduate or less	5,874	37.2 (34.8–39.7)	23,904	26.8 (25.7–27.9)	<0.001
Some college or more	14,002	33.9 (32.4–35.5)	47,050	23.0 (22.3–23.7)	<0.001
**Employment status**					
Employed or self-employed	9,574	25.9 (24.2–27.6)	35,168	15.5 (14.8–16.2)	<0.001
Unemployed	930	26.9 (21.9–32.6)	2,762	22.3 (19.5–25.3)	0.134
Unable to work	1,491	66.4 (61.2–71.2)	5,153	58.2 (55.3–60.9)	0.005
Retired	6,348	53.5 (51.1–55.9)	22,313	48.3 (47.0–49.7)	<0.001
Homemaker or student	1,435	26.0 (22.4–30.1)	5,320	11.7 (10.5–13.1)	<0.001
**Body mass index category^¶¶^**					
Underweight or normal	5,704	27.8 (25.5–30.1)	22,003	17.6 (16.6–18.5)	<0.001
Overweight	6,496	35.0 (32.7–37.4)	23,871	24.3 (23.3–25.5)	<0.001
Obese	6,619	42.4 (40.1–44.8)	20,976	33.4 (32.2–34.7)	<0.001
Physical inactivity***	4,876	42.2 (39.4–45.1)	20,268	31.5 (30.2–32.8)	<0.001
**State**					
Alaska	554	33.2 (27.4–39.5)	2,244	22.3 (19.8–25.1)	0.001
Hawaii	1,333	26.0 (23.1–29.0)	5,528	20.4 (19.1–21.8)	<0.001
Kansas	1,874	35.9 (33.3–38.7)	7,198	23.4 (22.3–24.6)	<0.001
Maine	1,056	37.1 (32.9–41.6)	4,169	30.2 (28.2–32.3)	0.005
Maryland	1,213	31.3 (27.8–35.1)	3,787	23.3 (21.6–25.1)	<0.001
Michigan	676	38.6 (34.1–43.3)	2,551	29.0 (26.9–31.1)	<0.001
New Jersey	1,051	31.6 (27.5–35.9)	3,994	23.4 (21.5–25.5)	<0.001
New Mexico	1,232	30.0 (28.6–33.7)	4,404	25.4 (23.7–27.1)	0.02
New York	816	30.9 (26.9–35.3)	2,998	21.9 (20.1–23.9)	<0.001
Ohio	801	44.3 (39.0–49.7)	2,780	28.5 (26.1–30.9)	<0.001
Oklahoma	654	35.4 (31.0–40.1)	2,153	25.5 (23.3–27.8)	<0.001
Oregon	1,082	36.0 (32.6–39.5)	4,170	25.5 (24.0–27.1)	<0.001
Rhode Island	1,090	33.5 (29.9–37.3)	3,801	26.9 (25.1–28.8)	0.002
Tennessee	1,271	39.0 (35.4–42.7)	3,650	30.6 (28.7–32.6)	<0.001
Texas	2,247	33.2 (29.6–36.9)	7,228	21.1 (19.5–22.8)	<0.001
Utah	1,155	33.3 (30.1–36.6)	4,128	20.4 (19.1–21.8)	<0.001
Virginia	1,805	36.7 (33.8–39.7)	6,428	26.2 (24.8–27.6)	<0.001

**Abbreviation:** BMI = body mass index.

* Having arthritis was defined as having ever been told by a doctor or other health care professional that the respondent had arthritis, rheumatoid arthritis, gout, lupus, or fibromyalgia.

† Caregiving was defined as providing care to a family member or friend with a health condition or disability during the past 30 days.

§ The following states implemented the arthritis and caregiving modules in the same survey year during 2017 or 2019 (most recent year used): Alaska (2017), Hawaii (2019), Kansas (2017), Maine (2019), Maryland (2019), Michigan (2017), New Jersey (2017), New Mexico (2017), New York (2019), Ohio (2019), Oklahoma (2017), Oregon (2019), Rhode Island (2017), Tennessee (2019), Texas (2019), Utah (2019), and Virginia (2019).

¶ Categories might not sum to the sample total because of missing responses.

** Estimates were weighted to each state’s adult population.

†† T-tests were used to determine statistically significant differences in arthritis prevalence between caregivers and noncaregivers for each subgroup of selected characteristics.

§§ Includes respondents who reported that they are of some other race group not listed in the survey question responses and are not of Hispanic origin.

¶¶ BMI (kg/m2) estimates were calculated from self-reported weight and height. BMI was categorized as underweight or healthy weight (BMI <25), overweight (BMI 25 to <30), and having obesity (BMI ≥30).

*** Physical inactivity was defined as responding “no” to the question, “During the past month, other than your regular job, did you participate in any physical activities or exercises such as running, calisthenics, golf, gardening, or walking for exercise?”

Compared with caregivers without arthritis, those with arthritis provided similar types of personal and household care and were more likely to have provided care for ≥5 years (35.1% versus 28.7%) and for ≥40 hours per week (20.9% versus 17.5%) (Table 2). Among adults with arthritis, 49.1% of caregivers reported arthritis-attributable activity limitations, and 39.9% of caregivers reported arthritis-attributable work limitations. Caregivers with arthritis were more likely than were those without arthritis to have the following types of disability: mobility (38.0% versus 7.3%), self-care (9.8% versus 1.5%), and independent living (14.7% versus 5.0%). Among caregivers with arthritis, 91.2% (95% CI = 89.8%–92.4%) reported having a primary care provider. Among caregivers who provided ≥40 hours of care per week, those with arthritis were more likely than those without arthritis to be unable to work (22.6% versus 7.6%) or to be retired (33.1% versus 18.4%) (Figure).

**TABLE 2:** Distribution of selected characteristics among caregivers* aged ≥18 years with and without arthritis^†^ — Behavioral Risk Factor Surveillance System, 17 states^§^, 2017 and 2019

Characteristic	Caregivers with arthritis		Caregivers without arthritis		p-value^††^
	Unweighted no.^¶^	Weighted** % (95% CI)	Unweighted no.^¶^	Weighted** % (95% CI)	
**Length of time of care provided, yrs**					
<5	5,256	64.9 (62.7–67.1)	8,121	71.3 (69.5–73.0)	<0.001
≥5	2,636	35.1 (32.9–37.3)	3,401	28.7 (27.0–30.5)	<0.001
**No. of hours of care provided weekly**					
<20	5,094	67.2 (64.9–69.3)	7,760	71.5 (69.7–73.2)	0.003
20–39	811	12.0 (10.4–13.7)	1,179	11.0 (9.8–12.2)	0.337
≥40	1,443	20.9 (19.1–22.8)	1,929	17.5 (16.1–19.0)	0.005
**Type of care provided^§§^**					
Personal care only	417	5.7 (4.8–6.8)	624	5.6 (4.8–6.5)	0.87
Household tasks only	2,621	32.7 (30.6–34.9)	3,910	34.7 (32.8–36.5)	0.17
Both types	3,350	44.8 (42.5–47.0)	5,005	43.8 (42.0–45.7)	0.54
Neither type	1,549	16.8 (15.3–18.4)	2,023	15.9 (14.6–17.2)	0.36
**Has a primary care provider^¶¶^**	7,464	91.2 (89.8–92.4)	9,916	80.4 (78.6–82.1)	<0.001
**Arthritis-attributable limitations**					
Has arthritis-attributable activity limitations***	3,884	49.1 (46.9–51.4)	NA	NA	NA
Has arthritis-attributable work limitations^†††^	2,802	39.9 (37.7–42.2)	NA	NA	NA
**Disability type^§§§^**					
Mobility	2,894	38.0 (35.8–40.2)	888	7.3 (6.4–8.3)	<0.001
Self-care	682	9.8 (8.5–11.3)	165	1.5 (1.1–1.9)	<0.001
Independent living	1,004	14.7 (13.1–16.4)	524	5.0 (4.2–5.8)	<0.001

**Abbreviation:** NA = not applicable.

* Caregiving was defined as providing care to a family member or friend with a health condition or disability during the past 30 days.

^†^ Having arthritis was defined as having ever been told by a doctor or other health care professional that the respondent had arthritis, rheumatoid arthritis, gout, lupus, or fibromyalgia.

^§^ The following states implemented the arthritis and caregiving modules in the same survey year during 2017 or 2019 (most recent year used): Alaska (2017), Hawaii (2019), Kansas (2017), Maine (2019), Maryland (2019), Michigan (2017), New Jersey (2017), New Mexico (2017), New York (2019), Ohio (2019), Oklahoma (2017), Oregon (2019), Rhode Island (2017), Tennessee (2019), Texas (2019), Utah (2019), and Virginia (2019).

^¶^ Categories might not sum to the sample total because of missing responses.

** Estimates were weighted to each state’s adult population.

^††^ T-tests were used to determine statistically significant differences in characteristics between respondents with and without arthritis.

^§§^ Personal care was defined as responding “yes” to the question, “In the past 30 days, did you provide care for this person by managing personal care such as giving medications, feeding, dressing, or bathing?” Household tasks was defined as responding “yes” to the question, “In the past 30 days, did you provide care for this person by managing household tasks such as cleaning, managing money, or preparing meals?”

^¶¶^ Having a primary care provider was defined as responding “yes,” “only one,” or “more than one” to the question, “Do you have one person you think of as your personal doctor or health care provider? (If ´No´ ask ´Is there more than one or is there no person who you think of as your personal doctor or health care provider?´).”

*** Arthritis-attributable activity limitation was defined as responding “yes” to the question, “Are you now limited in any way in any of your usual activities because of arthritis or joint symptoms?” This question was only asked among respondents with arthritis.

^†††^ Arthritis-attributable work limitations were defined as responding “yes” to the question, “Do arthritis or joint symptoms now affect whether you work, the type of work you do or the amount of work you do?” This question was only asked among respondents with arthritis.

^§§§^ Disability types were defined as responding “yes” to the following questions, “Do you have serious difficulty walking or climbing stairs?” (mobility disability), “Do you have difficulty dressing or bathing?” (self-care disability), and “Because of a physical, mental, or emotional condition, do you have difficulty doing errands alone such as visiting a doctor´s office or shopping?” (independent living disability).

**FIGURE:**
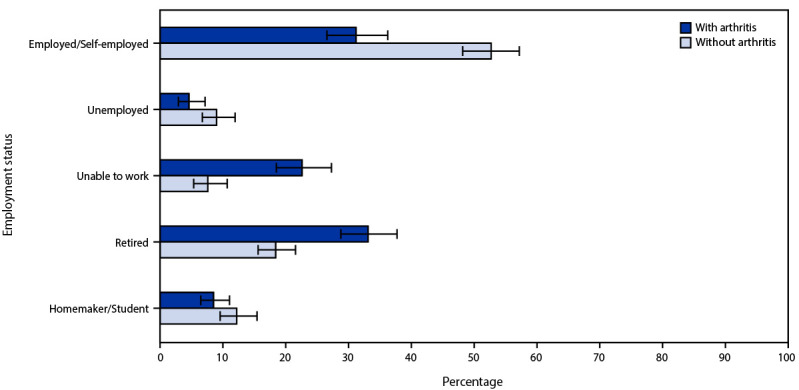
Employment status* of caregivers^†^ aged ≥18 years who provide ≥40 hours of care per week, by arthritis status^§^ — Behavioral Risk Factor Surveillance System, 17 states,^¶^ 2017 and 2019** * Determined based on the response to the question, “Are you currently employed for wages, self-employed, out of work for 1 year or more, out of work for less than 1 year, a homemaker, a student, retired, or unable to work?” ^†^ Caregiving was defined as providing care to a family member or friend with a health condition or disability during the past 30 days. ^§^ Having arthritis was defined as having ever been told by a doctor or other health care professional that the respondent had arthritis, rheumatoid arthritis, gout, lupus, or fibromyalgia. ^¶^ The following states implemented the arthritis and caregiving modules in the same survey year during 2017 or 2019 (most recent year used): Alaska (2017), Hawaii (2019), Kansas (2017), Maine (2019), Maryland (2019), Michigan (2017), New Jersey (2017), New Mexico (2017), New York (2019), Ohio (2019), Oklahoma (2017), Oregon (2019), Rhode Island (2017), Tennessee (2019), Texas (2019), Utah (2019), and Virginia (2019). ** Error bars represent 95% CIs.

## Discussion

Among adults in 17 states, one in five was a caregiver, and one in three caregivers reported arthritis. The prevalence of arthritis was higher among caregivers than among noncaregivers across nearly all demographic subgroups. Caregivers are critical members of the care team. As both the number of persons providing care for friends and family members (*1*) and the number of persons with arthritis increase (*5*), supporting caregivers with arthritis can help promote their own health along with the care they provide.

An estimated 49.1% of caregivers with arthritis reported arthritis-attributable activity limitations. Although not directly comparable, a previous report estimated that 43.9% (95% CI = 42.9%–44.8%) of adults with arthritis reported arthritis-attributable activity limitations during 2016–2018, suggesting that limitations specific to arthritis might be more common among caregivers than among the general population (*5*). In addition, caregivers with arthritis were more likely to have disabilities with mobility, self-care, and independent living than were caregivers without arthritis, and more than one in five caregivers with arthritis who provided ≥40 hours of care per week reported being unable to work. However, the types of personal and household tasks provided to the care recipient did not differ by arthritis status among caregivers, suggesting that such care might be necessary or expected of caregivers. Taken together, these findings suggest that caregivers with arthritis who have related disabilities and activity and work limitations might experience unique challenges to sustaining the care they provide, including financial insecurity because of loss of paid income (*6*).

A higher proportion of caregivers with arthritis also reported providing care for ≥40 hours per week and for ≥5 years than did caregivers without arthritis, suggesting that they might benefit from long-term services and supports. Ensuring that the health and well-being of all caregivers, including those with arthritis, is optimized can help them continue providing quality care. A large proportion of caregivers with arthritis reported having a primary care provider. These caregivers with arthritis can discuss their experiences with their health care provider and seek evidence-based programs for support, such as effective physical activity-based programs and self-management programs to help reduce arthritis symptoms and improve arthritis management and quality of life.^¶¶¶^^,^**** Caregivers can also learn more about ways to reduce their risk for developing arthritis or managing arthritis if they have it.^††††^

The findings in this report are subject to at least five limitations. First, because of the cross-sectional nature of BRFSS data, causality among caregiving, arthritis, and other conditions such as disability status cannot be determined. Second, self-reported data might be subject to several biases including recall and social desirability. Third, BRFSS data cannot be validated with medical records. Fourth, data were from 17 states and might not represent all jurisdictions. Finally, statistically significant differences in the prevalence of arthritis between caregivers and noncaregivers were not observed in some racial and ethnic groups, even though estimates were consistently higher among caregivers.

Caregiving is common in the United States, and many caregivers have arthritis and related limitations and disabilities. Caregivers with arthritis might benefit from interventions to help them continue providing quality care for their friends and family members. Health care professionals can recommend physical activity and lifestyle management programs for arthritis to help their patients who are caregivers to manage their arthritis symptoms.^§§§§^ Public health professionals can support all caregivers and care recipients by strengthening public health infrastructure using the public health strategist approach,^¶¶¶¶^ implementing strategies from the Healthy Brain Initiative and Building Our Largest Dementia (BOLD) Infrastructure Act for supporting caregivers,***** and accessing resources from the National Public Health Agenda for Osteoarthritis (*7*) and the BOLD Public Health Center of Excellence on Dementia Caregiving^†††††^(*8*).

**Summary**

**What is already known about this topic?**

Approximately 44% of adults with arthritis report arthritis-attributable limitations, but little is known about arthritis among caregivers.

**What is added by this report?**

During 2017 and 2019, one in five adults in 17 states was a caregiver, and one in three caregivers had arthritis. Prevalence of arthritis was higher among caregivers (35.1%) than among noncaregivers (24.5%). Compared with caregivers without arthritis, those with arthritis provided similar types of care and were more likely to have provided care for more hours per week and for more years and report having disabilities.

**What are the implications for public health practice?**

Arthritis among caregivers might affect the care they provide, which can be physically demanding. Health care professionals can support caregivers with arthritis and their care recipients by promoting arthritis-related health interventions.

## Footnotes

*
https://www.cdc.gov/arthritis/healthcare/index.html

†
https://www.cdc.gov/aging/healthybrain/issue-maps/supporting-caregivers.html

§ The following states implemented the arthritis and caregiving modules in the same survey year during 2017 or 2019 (most recent year used): Alaska (2017), Hawaii (2019), Kansas (2017), Maine (2019), Maryland (2019), Michigan (2017), New Jersey (2017), New Mexico (2017), New York (2019), Ohio (2019), Oklahoma (2017), Oregon (2019), Rhode Island (2017), Tennessee (2019), Texas (2019), Utah (2019), and Virginia (2019).

¶ https://www.cdc.gov/brfss/annual_data/2017/pdf/2017-response-rates-table-508.pdf; https://www.cdc.gov/brfss/annual_data/2019/pdf/2019-response-rates-table-508.pdf

** Determined based on the response to the question, “For how long have you provided care for that person? Would you say: less than 30 days, 1 month to less than 6 months, 6 months to less than 2 years, 2 years to less than 5 years, or more than 5 years?”

†† Determined based on the response to the question, “In an average week, how many hours do you provide care or assistance? Would you say: up to 8 hours per week, 9 to 19 hours per week, 20 to 39 hours per week, or 40 hours or more?”

§§ Determined based on the response to two questions regarding personal and household tasks. Personal care was defined as responding “yes” to the question, “In the past 30 days, did you provide care for this person by managing personal care such as giving medications, feeding, dressing, or bathing?” Household care was defined as responding “yes” to the question, “In the past 30 days, did you provide care for this person by managing household tasks such as cleaning, managing money, or preparing meals?”

¶¶
https://www.cdc.gov/brfss/data_documentation/pdf/BRFSS_Data_Users_Guide_on_Disability_Questions_2018-508.pdf

*** Arthritis-attributable activity limitations was defined as responding “yes” to the question, “Are you now limited in any way in any of your usual activities because of arthritis or joint symptoms?” This question was only asked among respondents with arthritis. Arthritis-attributable work limitations was defined as responding “yes” to the question, “Do arthritis or joint symptoms now affect whether you work, the type of work you do or the amount of work you do?” This question was only asked of respondents with arthritis.

††† Determined based on the response to the question, “Are you currently employed for wages, self-employed, out of work for 1 year or more, out of work for less than 1 year, a homemaker, a student, retired, or unable to work?”

§§§ 45 C.F.R. part 46.102(l)(2), 21 C.F.R. part 56; 42 U.S.C. Sect. 241(d); 5 U.S.C. Sect. 552a; 44 U.S.C. Sect. 3501 et seq.

¶¶¶
https://oaaction.unc.edu/aaebi/

****
https://www.cdc.gov/arthritis/interventions/index.htm

††††
https://www.cdc.gov/arthritis/basics/index.html

§§§§
https://www.cdc.gov/arthritis/healthcare/index.html

¶¶¶¶
https://www.cdc.gov/aging/caregiving/caregiver-brief.html

*****
https://www.cdc.gov/aging/healthybrain/issue-maps/supporting-caregivers.html

†††††
https://bolddementiacaregiving.org/
